# The emerging fungal pathogen *Cryptococcus gattii*: Epidemiology, pathogenesis, immunomodulatory attributes, and drug susceptibility

**DOI:** 10.1371/journal.pntd.0013245

**Published:** 2025-07-03

**Authors:** Chengjun Cao

**Affiliations:** College of Pharmaceutical Sciences, Southwest University, Chongqing, China; Universidad de Antioquia, COLOMBIA

## Abstract

The emerging fungal pathogen, *Cryptococcus gattii*, causes infections in both immunocompromised and immunocompetent individuals, often resulting in high mortality rates. While *Cryptococcus neoformans* is predominantly associated with cryptococcosis in immunocompromised patients, the significance of *C. gattii* infections has garnered attention due to its prevalence among seemingly healthy individuals. Notably, *C. gattii* exhibits distinct epidemiological patterns, geographic distribution, genotypes, and phenotypes compared to *C. neoformans*. However, the comprehension of *C. gattii*’s virulence characteristics, regulatory mechanisms, and therapeutic avenues has lagged behind those of *C. neoformans*. The less robust clinical and epidemiological data, coupled with the limitations of effective treatment options, underscore the urgency in addressing *C. gattii* as a serious public health threat. In this review, I discuss the epidemiology, virulence factors, regulatory mechanisms, immunomodulatory attributes, and drug susceptibility of *C. gattii*. This comprehensive discussion aims to enhance our understanding of this emerging fungal pathogen and potentially contribute to the development of more effective prevention and management strategies.

## Introduction

*Cryptococcus* species, notably *Cryptococcus neoformans* and *Cryptococcus gattii*, are major causes of life-threatening fungal meningitis [1]. These two species complexes exhibit distinct epidemiological patterns: *C. neoformans* predominantly affects immunosuppressed individuals, whereas *C. gattii* has been observed to cause cryptococcosis in both immunocompromised patients and those without apparent immune deficiency [2,3]. *C. gattii* has been recognized as an emerging fungal pathogen with an expanding environmental niche [4]. Within the *C. gattii* species, isolates can be further subdivided into two serotypes (B and C) and six genotypes (VGI, VGII, VGIII, VGIV, VGV, and VGVI), facilitated by advanced molecular and phenotypic analyses [2]. These geographical distinctions among the VG lineages reflect the intricate microevolutionary patterns within the *C. gattii* species complex (CGSC) over time. These ongoing processes underscore the continuous evolution of the CGSC, ensuring its adaptation to diverse environmental niches. Infectious propagules of *C. gattii* were inhaled into the lungs to establish fungal infection. Several virulence factors and signaling pathways that contribute to the pathogenicity of *C. gattii* have been identified. Alarmingly, the absence of dedicated antifungal drug trials to guide treatment of *C. gattii* infections underscores the urgency for effective therapeutic strategies. This environmental fungus has garnered global attention due to its increasing prevalence and associated high morbidity and mortality rates. Thus, a comprehensive understanding of its pathogenic characteristics and the development of targeted therapeutic interventions are vital in addressing the challenge posed by *C. gattii* infections.

## Methods

To prepare a review on the emerging fungal pathogen *Cryptococcus gattii*, the keywords “*Cryptococcus gattii*” and “fungal virulence” were used to identify relevant articles published from 2010 to 2024 using the PubMed and ScienceDirect databases. The search was limited to English-language articles. Relevance was assessed through title and abstract, and selected full-text articles were reviewed based on the information regarding the epidemiological patterns, virulence factors, molecular regulation pathways, the immune response, and drug susceptibility of *C. gattii*. Additional references were identified through citation tracking of selected articles.

## Epidemiology

Recent estimates indicate that cryptococcal meningitis affects more than 190,000 individuals, resulting in approximately 150,000 fatalities worldwide annually [1]. Prior to the advent of acquired immunodeficiency syndrome (AIDS), the incidence of cryptococcosis was relatively low. However, the HIV pandemic has dramatically reshaped the landscape of *Cryptococcus* infections, with cryptococcosis accounting for approximately 19% of HIV/AIDS-related deaths [5]. *C. neoformans* is the leading cause of cryptococcosis in immunocompromised individuals, particularly those afflicted by HIV. While the vast majority of *C. gattii*-infected patients are immunocompetent and apparently healthy individuals without HIV infection [2,6]. The VGI genotype prevails globally in both environmental and clinical settings, serving as the most common isolate [7]. Outbreaks on Vancouver Island and the Pacific Northwest (PNW) have been traced primarily to the VGII genotype. When considering the high global incidence rates of *C. gattii* infections, genotypes VGI and VGII are the primary *C. gattii* pathogens among non-AIDS patients [8]. Lineages VGIII and VGIV are dominant *C. gattii* types associated with AIDS patients [9].

*C. gattii* has the capacity to infect humans as well as domestic, terrestrial, and marine animals [10]. Cryptococcosis is not contagious. Both humans and animals can be infected by inhalation of desiccated yeast cells or basidiospores from the environment [10]. Increased environmental exposure is a significant reason for *C. gattii* infection [11]. The ability of healthy individuals’ host defense systems to eliminate fungal pathogens ensures that only a fraction of the exposed population ultimately develops *C. gattii* infection. However, predisposing risk factors significantly contribute to this outcome. These include the presence of autoantibodies against granulocyte-macrophage colony-stimulating factor (GM-CSF), preexisting use of oral steroid use, a history of cancer or chronic lung disease, and the virulence of the *C. gattii* genotype [9].

## Virulence factors

**Fig 1:**
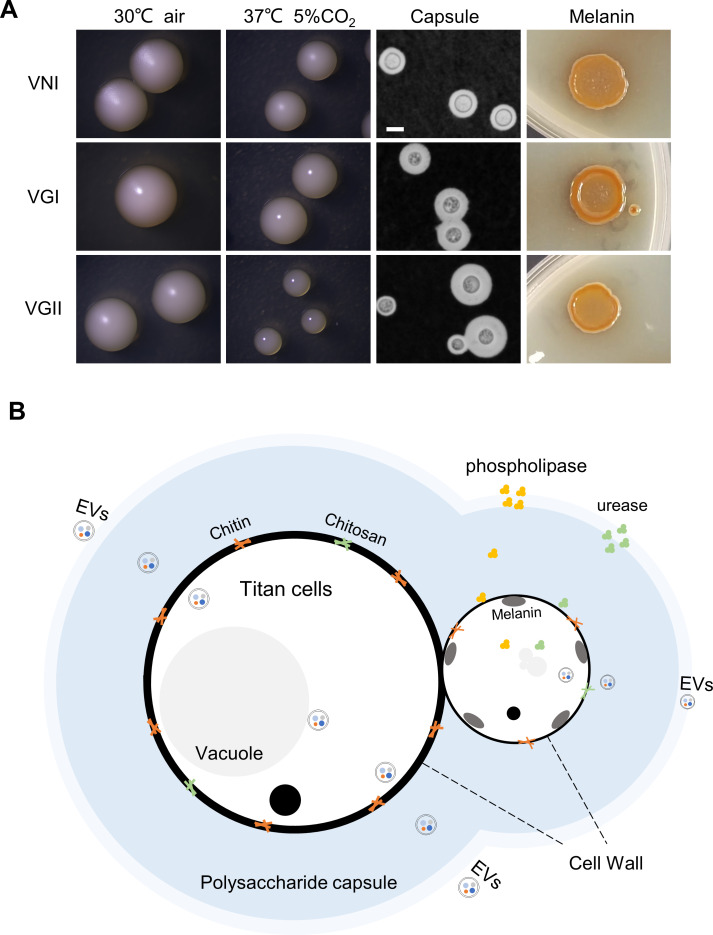
Virulence factors identified in C. *gattii.* **(A)** Host conditions fitness, capsule and melanin production of *C. gattii* (VGI and VGII genotypes) and *C. neoformans* (VNI genotype). Growth assays at 30° in the air, at 37° in 5% CO_2_. India ink staining under light microscopy reveals the capsule. Melanin production at 30° on Niger seed medium. Bar, 5 μm. **(B)** Virulence factors associated with fungal pathogenesis and function in *C. gattii.* These virulence factors include polysaccharide capsule, melanin, cell wall components such as chitin and chitosan, titan cells, extracellular vesicles (EVs), and extracellular enzymes.

The classic cryptococcal virulence factors, such as the polysaccharide capsule, melanin, and the ability to grow at physiological temperature, are involved in the pathogenicity of *C. neoformans* and have been reviewed in detail [2]*.* Although these virulence strategies are shared by pathogenic *C. gattii*, there are some crucial differences between the two species complexes (Fig 1).

### Genotypes

Different lineages of CGSC have been isolated from patients, VGIII and VGIV infections occur mainly in HIV/AIDS patients, whereas VGI and VGII were isolated in immunocompetent hosts [12–14]. Survival data using a *Drosophila* infection model found that VGIII was the most virulent molecular type [15]. Studies on the Vancouver Island outbreak strains showed the virulence difference within VGII that the subtype VGIIa is highly virulent compared to VGIIb based on a murine infection model [16]. These findings point to the influence of molecular subtypes on fungal virulence of *C. gattii*. However, an intriguing complexity arises when considering the virulence spectrum across different isolates within each major molecular type. *Galleria mellonella* infection studies demonstrate that *C. gattii* strains, regardless of their molecular types, encompass a broad range of virulence capabilities [14,17]. The coexistence of both low and highly virulent strains within all major molecular types suggests that virulence is not solely determined by molecular type but influenced by additional virulence factors.

### Capsule

The polysaccharide capsule of *Cryptococcus* is composed of a major glucuronoxylomannan (GXM, formed by mannose backbone with xylose and glucuronic acid side chains) and two minor galactoxylomannan (GXMGal, galactan backbone with galactomannan side chains that are further substituted with variable numbers of xylose residues) and mannoproteins [18]. The GXM structures of *C. gattii* and *C*. *neoformans* differ in the degree of mannose backbone substitution, which can divide cryptococcal strains into different serotypes (A, B, C, D, and AD) [18].

The function and structure of the capsule have been well studied and documented in *C*. *neoformans*. The polysaccharide capsule is critical for host infection of *C. neoformans* [2,18]. During lung invasion, the capsule undergoes dramatic expansion and density modulation, a phenomenon intricately influenced by host factors like serum, high CO_2_ concentration, low iron, and nutritional deprivation. The capsule provides a physical barrier that protects the fungus from desiccation and phagocytic predators, such as amoeba [18]. The enlarged capsule confers resistance to oxidative stresses to protect yeast cells against host phagocytic cells [18]. However, the sole reliance on capsule size as a virulence biomarker is tenuous, given the conflicting findings across various studies. Therefore, the relationship between capsule size and fungal virulence remains a subject of ongoing investigation.

The capsule function of *C*. *gattii* is different from *C*. *neoformans*, which was discovered when comparing the virulence difference between an acapsular cryptococcal mutant strain coated with capsular extract from *C*. *gattii* and from *C*. *neoformans* [19]. Although the fungal biology of the capsule is likely to be conserved in *C. gattii*, the impact of capsule composition on *C. gattii* virulence remains unclear. Acapsular yeast cells of *C. gattii* were virulence attenuated in a mouse infection model, indicating that the capsule of *C. gattii* is required for pathogenicity and evasion from the host immune system [20,21]. The relationship between capsule size and fungal virulence is further complicated by observations that exposure of *C. gattii* to agrochemical benomyl and antifungals can diminish capsule size without compromising fungal virulence [22]. Thus, capsule size and structure play important and complex roles in *C. gattii*.

### Melanin

The production of melanin pigment serves as a pivotal virulence factor of cryptococci, playing a crucial role in the pathogenicity of these fungi [23]. The dark macromolecular melanin maintains cell wall integrity and protects fungal cells against environmental stressors, including ultraviolet and ionizing radiation, as well as oxidative stress [23]. Laccase catalyzes the biosynthesis of melanin in the presence of phenolic compounds, such as L-3,4-dihydroxyphenylalanine (L-DOPA), dopamine, epinephrine, norepinephrine and caffeic acid [24]. Notably, neurotransmitters can also serve as substrates to produce melanin during host infection in *Cryptococcus* species [24], indicating the association of melanin production with the central nervous system (CNS) infection. Moreover, the expression of laccase promotes intracellular proliferation in macrophages, while laccase-deficient mutant displays attenuated pulmonary dissemination [12,25], highlighting the enzyme’s significance in virulence. It has been reported that the ability of melanin production was directly associated with fungal virulence in *C. gattii* in a *G. mellonella* infection model [14]. Melanin production protects against reactive oxygen species and evades the host immune system, possibly correlating with fungal virulence and subsequent morbidity and mortality.

### Chitin and chitosan

The fungal cell wall is an essential structure that provides the primary barrier against host defenses. Chitin and chitosan are important components in the cell wall that significantly modulate the interplay between host and pathogen. Chitosan-deficient strains of *C. neoformans* induce robust host immune responses during infection while exhibiting attenuated virulence, indicating the important role of chitin and chitosan in *Cryptococcus* virulence [26]. Whole-genome transcriptome analyses of *C. gattii* and *C. neoformans* have shown notable differences in expression levels of genes involved in chitin and chitosan biosynthesis, suggesting the transcriptional regulation divergence of chitin and chitosan biosynthesis between the two species [27,28]. Notably, *C. gattii* R265 cells have been found to produce a substantially larger amount of chitosan in their cell wall during host infection compared to *C. neoformans* KN99 [28]. Intriguingly, despite no discernible impact on melanin production, a chitin deacetylase mutant *cda3*Δ exhibits a notable virulence defect in the murine intranasal infection model [28], further emphasizing the crucial role of cell wall chitin and chitosan in facilitating *C. gattii* infection.

### Biofilms

Biofilms, highly structured microbial communities enclosed in an extracellular polymeric matrix, are ubiquitous among microorganisms in nature and provide various adaptive attributes for microorganisms, including increasing the concentration of nutrients, enhancing cell-to-cell interactions, and stress resistance [29]. When biofilms form on implanted biomedical devices, they pose significant challenges, leading to device dysfunction, antimicrobial resistance, and host defense. Some strains of *C. gattii* have a great ability to form highly organized and complex biofilm on abiotic surfaces like polyvinyl chloride and silicone catheter [29]. Yeast cells adhere to the substratum in a monolayer in the early stage of biofilm formation. Then, *C. gattii* cells produce extracellular fibrils to connect yeast cells and the abiotic surface until the mature biofilm formation [29]. Transcriptomic profiling of *C. gattii* biofilm formation on polystyrene surfaces, as compared to free-floating planktonic cells, has unveiled differential gene expression patterns. These changes encompass metabolic pathways, information processing mechanisms, stress response systems, and cell-to-cell adhesion factors [29]. The comparative proteome of *C. gattii* grown under planktonic and biofilm conditions found that up-regulated proteins are related to oxidative stress, mitochondrial electron transport, metabolic process-related proteins, and transcription, aligning with prior transcriptomic findings [30]. The biofilm’s adaptation to utilize alternative carbon sources enhances fungus survival fitness during infection. Additionally, the biological processes enriched in the biofilm proteome exhibit similarities to those observed in the transcriptome of *C. gattii* R265 recovered from bronchoalveolar lavage of infected mice [31], suggesting shared pathways underpinning both biofilm development and lung infection by *C. gattii*. The ability of forming biofilms has been associated with fungal virulence, as evidenced by the heightened virulence displayed by the biofilm-forming *C. gattii* cells compared to their planktonic counterparts in the *G. mellonella* infection model. Furthermore, this biofilm formation is correlated with significant histopathological damage to pulmonary tissues observed in animal infection models, underscoring its pivotal role in disease pathogenesis [32,33].

### Cell gigantism

In addition to capsule structure and size change that occur during lung infections of *Cryptococcus* species, cellular size heterogeneity has been recognized as an important virulence factor during infection [34–36]. Enlarged cryptococcal cells, also known as giant or titan cells, were observed in clinical specimens and have been largely studied in *Cryptococcus* [37]. The defining attributes and regulatory mechanisms of titan cells have been predominantly elucidated in *C. neoformans* [37]. The important phenotypical feature of titan cells is their huge size, far exceeding the size of typical cells, which are 5–8 µm in diameter. Typical cells are 5–8 µm in diameter, and the cell body sizes of titan cells range from 25 to 30 µm in diameter, with some reaching extraordinary dimensions of up to 100 µm in diameter in the lungs of infected mice [37]. Titan cells isolated from infected lungs exhibit more phenotypical characteristics, including a single nucleus with an augmented genome copy number, a thicker cell wall, and a large vacuole. The enlarged titan cells confer protection of the entire population of cryptococcal cells from phagocytosis and play a critical role in establishing pulmonary infection [37].

Examination of phenotypic variation of cryptococcal isolates from HIV/AIDS patients in Botswana indicated that *C. gattii* seems to have a greater propensity to form titan cells than *C. neoformans* in response to a host-relevant environment [38]. During *G. mellonella* infection, the cell size of all *C. gattii* strains studied underwent significant enlargement, while cells grown under nutrient conditions exhibited normal cell body size, indicating the involvement of cell size enlargement during infection [14]. To understand the biology and mechanism underlying titanization, *in vitro* titan induction systems have been performed in *C. gattii* [39,40]. The capacity to form titan cells in *C*. *gattii* strains was significantly higher than in *C*. *neoformans* and all *C*. *gattii* isolates (VGI–VGIV) tested were able to form titan cells in the *in vitro* conditions. Cell enlargement is asynchronous with DNA replication. The polyploid titan cells are unbudded in *C. gattii* R265, which differs from *C. neoformans* in that titan cells undergo cell division to produce normal-sized daughter cells [40]. This difference may explain the lower ability of cell dissemination outside the lungs in *C. gattii* compared to *C. neoformans*.

### The ability to grow at physiological temperature and CO_2_ level

Upon host infection, *Cryptococcus* species must adapt to various host-specific stress conditions, such as higher physiological temperature, higher carbon dioxide (CO_2_) concentration, and nutrient limitation. High-temperature growth is one of the canonical virulence factors of *C*. *gattii* and *C*. *neoformans*. Although the ability to grow at a physiological temperature or higher alone is insufficient to cause disease in mammals, it is essential for invasive infection [41]. Gene deletions with growth defects under high temperatures also exhibit virulence attenuation [42]. The calcineurin pathway is critical for cryptococcal growth at 37 °C, and mutant strains disrupting the calcineurin genes are avirulent in *C. neoformans* and *C. gattii* [43,44]. The strain R265 lacking calcineurin function is thermotolerant but shows virulence defect [44], indicating that high-temperature tolerance is not sufficient but necessary for fungal virulence.

The CO_2_ concentration is very low (approximately 0.04%) in the environment. In contrast, the CO_2_ levels in mammalian host tissues range from 4.5% to 30%. Environmental isolates of *C. neoformans* have reduced fungal virulence compared to clinical isolates, even though strains have similar ability to form canonical virulence factors (physiological temperature growth, melanization, and capsule production). The ability to grow in higher levels of CO_2_ is different between environmental and clinical isolates and can also be used to distinguish fungal virulence during host infection [45]. Additionally, CO_2_ tolerance is important for antifungal drug susceptibility and dissemination of *C. neoformans* from the lung to the brain [46,47]. The ability to grow in the host levels of CO_2_ has been recognized as a virulence trait for *C. neoformans*. High CO_2_ levels promote titan cells and polysaccharide capsule formation in *C. gattii* [39,40], indicating the role of CO_2_ tolerance in the virulence of *C. gattii*, although it has not been directly investigated.

### Extracellular vesicles

Extracellular vesicles (EVs) are bilayered lipid membranous structures that export molecules from cells to the extracellular milieu [48]. Components characterized in cryptococcal EVs contain ribosomal proteins, proteins associated with virulence and stress response, capsular GXM, laccase, urease, and RNAs. Thus, they have been termed as ‘virulence bags’ [49]. As facultative intracellular pathogens, Cryptococcus species utilize EV-boosted phagocytosis to offer survival advantages in the host. *C. gattii* EVs with significantly increased GXM concentration were detected in the mutant with increased capsule size [50]. Analysis of the small molecule (molecular mass <900 Daltons) composition of EVs obtained from solid cultures of *C. gattii* revealed lots of previously unknown components. One of the vesicular peptides protects *G. mellonella* from *Cryptococcus* infection, indicating the potential of extracellular peptides produced by *C. gattii* as a fungal vaccine [51]. Although the role of *C*. *neoformans-*derived EVs could potentially be applied to *C*. *gattii*, distinctive features of EVs, including EV size, protein preference, and function in the two species complexes, have been identified [19,49]. *C. gattii* EVs isolated from virulent strains accumulate in the phagosomes and trigger intracellular growth of hypovirulent strains [52]. This discovery of *C. gattii* EV-based pathogen-to-pathogen communication and coordination has not been reported in *C*. *neoformans*. *C. gattii*-specific EV features broaden our knowledge of cryptococcal EVs and indicate the diversity of pathogenic functions.

## Signaling pathways involved in fungal virulence of *C. gattii*

**Fig 2:**
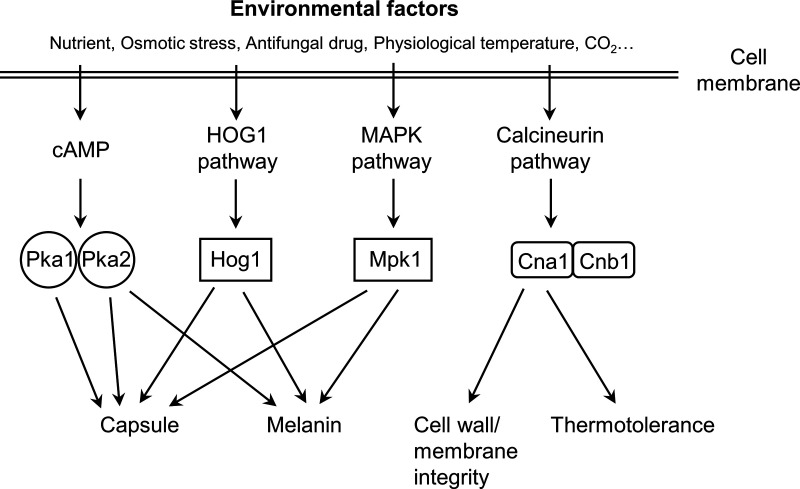
Signaling pathways involved in fungal virulence in *C. gattii.* The conserved signaling pathways, such as the cyclic AMP (cAMP)/protein kinase A (PKA), high osmolarity glycerol response (HOG), MAPK, and calcineurin pathways, play critical roles in the stress responses and virulence factors production of *C. gattii.*

The conserved signaling pathways, such as the cyclic AMP (cAMP)/protein kinase A (PKA), high osmolarity glycerol response (HOG), calcineurin, and cell wall integrity pathways, play critical roles in the stress responses and virulence factors production of *C. gattii* (Fig 2).

cAMP functions as the second messenger-mediated PKA signaling pathway that is critical in the pathogenicity and morphological differentiation of fungal pathogens. The cAMP/PKA pathway responds to a variety of environmental cues, such as higher CO_2_ levels, glucose starvation, low nitrogen, and methionine, to regulate melanin and capsule biosynthesis, titan cell production, environmental stress response, and mating process in *C. neoformans* [37,53,54]. PKA is also involved in the formation of virulence factors in *C. gattii*. Identification of the role of Pka1 and Pka2 catalytic subunits in melanin and capsule production found that both Pkas share redundant roles in regulating capsule production, and only Pka2 is responsible for melanin formation [55].

The high-osmolarity glycerol mitogen-activated protein kinase (HOG-MAPK) pathway has a notable effect on environmental stress adaptation and is essential for virulence regulation and sexual differentiation of *C. neoformans* [56,57]. The regulatory mechanism of Hog1, a key component of the HOG1-MAPK pathway, seems differ between *C. neoformans* and other fungi in the phosphorylation state under stress conditions. Hog1 of most *C. neoformans* isolates is highly phosphorylated under normal conditions and dephosphorylated in response to various deleterious stimuli such as osmotic stress, oxidative response, high ion concentration, and high temperature [57–59], which in contrast to other fungi (such as *Saccharomyces cerevisiae*) that Hog1 undergoes phosphorylation under stress conditions [60]. While the phosphorylation state of Hog1 has been studied in *C. neoformans*, similar investigations in *C. gattii* remain unclear. It has been characterized that Hog1 is involved in the stress response and fungal virulence in *C. gattii* [59]. Similar as *C. neoformans*, Hog 1 positively regulates stresses responses in *C. gattii*. Deletion of *HOG1* decreases capsule production, melanin synthesis, and fungal virulence in *C. gattii* [59]. However, Hog1-mediated control of capsule and melanin production is distinct in different serotypes of *C. neoformans* [61]. These observations suggest that *HOG1* has developed distinctive virulence regulatory mechanisms in the two *Cryptococcus* species and provides an understanding of the pathogenesis of *C. gattii*.

The Ca^2+^/calcineurin pathway responds to environmental signals, including thermotolerance, high CO_2_, and alkaline pH [60,62]. External cues elevate intracellular Ca^2+^ levels by extracellular Ca^2+^ transportation and internal pooled Ca^2+^ release. Increased Ca^2+^ binds to calmodulin and activates serine/threonine protein phosphatase, calcineurin. The roles of *C. gattii* calcineurin have been characterized in different molecular types, and studies found that VGIIa isolates were more tolerant to 37 °C in the presence of calcineurin inhibitors compared to isolates of other molecular types. However, the *cna1*Δ mutant of VGIIa isolate exhibited a clear growth defect at 37 °C, indicating that calcineurin is critical for thermotolerance and fungal virulence of *C. gattii* [44]. In addition, *C. gattii* calcineurin positively regulates cell membrane and cell wall integrity, as well as in ER stress responses may also contribute to its pathogenicity.

Additional genes linked to the virulence of *C. gattii* have been identified and reviewed in detail [12,25,63]. Deleting the signal transduction pathway molecule Mpk1 causes defect of melanin production, cell wall integrity, and fungal virulence in *C. gattii*. Superoxide dismutase Sod1 and Sod2 are required to produce virulence factors and fungal virulence during mouse inhalation or intravenous inoculation. Tps1 and Tps2 are critical for thermotolerance, capsule and melanin production, and pathogenicity in *C. gattii*. Transcription factor Ste12α regulates melanin, mating, virulence, and ecological fitness of *C. gattii*. The GATA transcription factor Gat2 plays an important role in the virulence in *C. gattii* but not in *C. neoformans* during infection in the mouse intrapharyngeal instillation model. Other genes involved in *C. gattii* virulence include D-amino acid oxidase genes (*DAO2*) [64], β-carbonic anhydrase (*CAN2*) [65], *CAP59* and *CAP60* [66], zinc finger proteins (*ZAP1, ZAP2,* and *ZIP3*) [67–69], Deubiquitinase Ubp5 [42], chitin deacetylases (*CDA3*) [28], small heat shock protein Hsp12.1, and candidate genes identified by microarray and transcriptional analysis [31,70].

## Immunomodulatory attributes of *C. gattii*

The ability of *C. gattii* to infect apparently immunocompetent individuals suggests a distinct immune response of the host to this fungal pathogen compared to *C. neoformans*. The innate immune system constitutes the first line of defense against cryptococcal infection, and an adaptive immune response is critical for the control of disease progression.

Lung-resident macrophages are the first host immune cells that interact with inhaled fungi [71]. GXM of cryptococcal polysaccharides can activate the toll-like receptor (TLR)-mediated innate immune response [72,73]. GXM samples from *C. gattii* strains induced nitric oxide (NO) production by RAW264.7 macrophages, while *C. neoformans* GXM did not [73]. Dectin-3, a member of the C-type lectin receptors (CLRs) family, has been identified as a direct receptor for the capsular GXM from *C. gattii* serotype B, but not *C. gattii* serotype C. Comparative studies between bone marrow-derived macrophages (BMDMs) from wild-type (WT, *Clec4d*^+*/*+^) and dectin-3-deficient (*Clec*^−*/*−^) mice found that the nuclear translocation of NF-κB p65 subunit, phosphorylation of IκBα, degradation and phosphorylation of extracellular signal-regulated protein kinase (ERK), and the secretion of pro-inflammatory cytokines such as tumor necrosis factor-alpha (TNF-α) and interleukin 6 (IL-6) production were different when exposed to GXM. These observations indicate that GXM of *C. gattii* serotype B are recognized by dectin-3 and mediated the activation of NF-κB and ERK pathways [74]. Caspase recruitment domain family member 9 (CARD9) is an adaptor protein that functions downstream of CLRs, facilitating the activation of NF-κB and ERK pathways [75]. Dectin-3- or CARD9-deficient mice infected with *C. gattii* had decreased survival and increased fungal burden in the lung due to impairment of alveolar macrophage activation [74]. Notably, variations in genotype among *Cryptococcus* strains impact their immunological properties, necessitating further inquiry into whether dectin-3 plays a role in recognizing diverse genotypes of *C. gattii*.

During the activation of adaptive immune responses to pathogens, dendritic cells (DCs) are the most efficient antigen-presenting cells (APCs) that present antigens to T cells. *C. gattii* is capable of evading host cell-mediated immune defenses initiated by DCs. *C. gattii* is killed by DCs, but failed to induce DCs maturation, leading to defective T cell responses [76]. The capsule of *C. gattii* masks an essential ligand that associates and activates DC surface receptor and is involved in the process of DC maturation, which is sufficient to recover T cell responses against *C. gattii* [77]. Additionally, *C. gattii* can escape innate immune responses by altering capsule structure since purified GXM from *C. gattii* produced lower levels of inflammatory cytokines from DCs, and an acapsular *cap60*Δ mutant was easily phagocytosed and killed by DCs [20,21]. Further study found that sustained filamentous actin (F-actin) formed a cage-like structure to conceal the phagosomes from recognition and block their maturation to phagolysosomes. Phagosomal maturation is essential for intracellular fungal killing by DCs and subsequent antigen processing and presentation [78]. These observations reveal a unique mechanism of DC immune evasion that permits *C. gattii* infection in immunocompetent individuals.

*C. gattii* causes different cytokine responses in the host when compared to *C. neoformans*. The cytokine profile of human peripheral blood mononuclear cells (PBMCs) of healthy individuals after being stimulated with heat-killed isolates of two *Cryptococcus* species was analyzed. Clinical *C. gattii* isolates induce higher levels of pro-inflammatory cytokines such as IL-1β, TNF-α, and IL-6 and the T-cell cytokines IL-17 and IL-22 than that of *C. neoformans* [79]. The examination of peripheral blood transcriptional changes in response to infection with *C. gattii* and *C. neoformans* in a mouse infection model found that components of the classical complement activation pathway and genes involved in M1 macrophage activation are up-regulated in *C. gattii*, indicating the alternative Th2-activated M2 macrophage polarization [80,81]. The cytokine types depend on the recognition of microbial components presented in the cells of the innate immune system. TLR4 and TLR9, but not TLR2, are pattern recognition receptors (PRRs) involved in the host’s cytokine response to *C. gattii*, suggesting the different pathogen-associated molecules on the cell surface of *C. gattii* and *C. neoformans* since TLR2 is known to recognize chitin in *C. neoformans* [82,83].

*C. gattii* and *C. neoformans* have different preferences for organ colonization that *C. gattii* is more likely to cause pulmonary infection, and the central nervous system is the primary target organ of *C. neoformans* [2]. The *C. gattii* strains suppress the protective immune responses by inhibiting the migration of neutrophils to the lungs of infected mice compared to *C. neoformans* infection [84,85]. In addition, the study of *C. gattii* strains showed lower levels of neutrophil infiltration and reduced cytokine production in the lungs of *C. gattii*-infected mice compared to those of mice infected with *C. neoformans* [85]. The study addresses the impact of *C. gattii* infection on the development of adaptive T helper cell immune response and found that fewer pulmonary Th1 and Th17 cells could be detected in mice infected with *C. gattii* strains when compared to *C. neoformans* infection. The expression levels of Th1-attracting chemokines were significantly reduced in the lungs of virulent *C. gattii*-infected mice. DCs in *C. gattii*-infected mice failed to induce effective Th1 and Th17 differentiation, suggesting that *C. gattii* infection diminished the DC-mediated protective Th1/Th17 immune responses [86].

The screening of the plasma samples of “apparently immunocompetent” patients with meningoencephalitis found that granulocyte-macrophage colony-stimulating factor (GM-CSF) neutralizing antibodies may be a risk factor for infection of *C. gattii* in HIV-negative and apparently healthy individuals. GM-CSF is a family of glycoprotein cytokines essential for the differentiation of monocytes to macrophages and modulating the immune response [11].

High titers of neutralizing anti-GM-CSF Abs were identified in 15 patients with cryptococcosis (15/39, 38.5%). Over 90% of GM-CSF autoantibodies-positive cryptococcosis clinical cases had central nervous system infection. The majority of these patients were confirmed to be infected with *C. gattii* [87]. GM-CSF autoantibodies are more strongly associated with *C. gattii* infection than with *C. neoformans* infection, which explains the difference in host immune responses between the two *Cryptococcus* species.

## Antifungal susceptibility

A limited number of antifungal drugs are available for the treatment of cryptococcosis. The most important treatment options for cryptococcosis are fluconazole, amphotericin B, and flucytosine [88]. Both *C. neoformans* and *C. gattii* are resistant to echinocandin class of drugs [88]. Clinical outcomes of fungal disease are influenced by multiple factors, including host immune status and treatment compliance, fungal virulence, drug characteristics, and antifungal susceptibility [2,6]. The *in vitro* antifungal susceptibility is related to the outcome of cryptococcal infection, especially in patients failing to respond to antifungal therapy [88]. These susceptibility testing methods provide microbiologic techniques and laboratory standardization to predict clinical outcomes based on *in vitro* data.

Different antifungal susceptibility profiles have been reported among cryptococcal species [89–91]. In general, several studies have reported that *C. gattii* isolates show comparatively low MICs of standard antifungals compared with those of *C. neoformans* [25,92]. Azoles are the most commonly used drugs to treat cryptococcal infections. Antifungal susceptibility assays showed that *C. gattii* isolates have the same MIC value for itraconazole as *C. neoformans* isolates, but were less susceptible to fluconazole and voriconazole than *C. neoformans* isolates [92]. An antifungal susceptibility study including both clinical and environmental isolates showed that *C. gattii* strains were less susceptible than *C. neoformans* to azoles but not amphotericin B and 5FC [93]. *In vitro* antifungal susceptibility of *Cryptococcus* isolates from Brazil showed higher MIC values for fluconazole, voriconazole, amphotericin B, and 5FC to *C. gattii* than for *C. neoformans* [94].

The different patterns of antifungal susceptibility within isolates of *C. gattii* have also been documented [95–98]. *C. gattii* from Taiwan showed comparable MICs of antifungal agents (itraconazole, fluconazole, voriconazole, posaconazole, flucytosine, and amphotericin B) against isolates from environmental and clinical samples. Significant differences in antifungal susceptibility among *C. gattii* strains of different genotypes were detected, and strains of VGI were more susceptible to azoles and flucytosine compared with strains of VGII [95]. Antifungal susceptibility of clinical *C. gattii* isolates from Colombia varies among molecular types VGI, VGII, and VGIII. VGI and VGII were less susceptible to 5FC and azoles, respectively than other molecular types [96]. However, the lack of data on clinical outcomes relative to MICs has prevented the establishment of clinical interpretive breakpoints (CBPs) for *Cryptococcus* species. Thus, *in vitro* susceptibility testing based on MICs is not currently recommended for the treatment of cryptococcosis.

*C. gattii* frequently resists limited antifungal drugs, especially azoles, because of long-term azole therapies for fungal disease treatment. Studies have examined the underlying mechanisms of antifungal drug resistance [92,99,100]. Azoles interact with the lanosterol 14-α-demethylase, encoded by the *ERG11* gene, resulting in cell membrane integrity defect of fungal pathogens [101]. The role of the *ERG11* gene in azole resistance of clinical isolates of *C. gattii* with higher MICs from the PNW has been investigated, and the study found that neither *ERG11* overexpression nor mutations in *ERG11* coding sequences contribute to the high azole MICs observed [102]. The antifungal susceptibility profile of Colombian clinical isolates of *C. neoformans* and *C. gattii* to fluconazole, voriconazole, and itraconazole showed that *C. gattii* isolates were less susceptible to azoles than *C. neoformans* isolates, which indicates that differences in the amino acid composition and structure of *ERG11* of the two species correlated with the MIC difference. Based on the analysis of the *ERG11* gene sequences of clinical isolates of both *C. neoformans* and *C. gattii,* a G973T mutation resulting in the substitution R258L was identified in the substrate recognition site of *ERG11* in a *C. gattii* isolate with high MICs for fluconazole and voriconazole [92]. The molecular mechanisms of differences in azole susceptibility in different subtypes of *C. gattii* have been investigated using clinical *C. gattii* strains comprising 5 VGI subtypes and 3 VGII subtypes. Similar to previous reports, VGI showed lower MICs compared to VGII [95,97,102]. Furthermore, analysis of transcriptional profiles of VGI and VGII strains found that genes related to transporter activities were differentially expressed, and ABC transporter genes were significantly higher expressed in VGII strains than in VGI strains [100], indicating the involvement of ABC transporter in azole sensitivity in *C. gattii*. Deletion of *FCY2* in *C. gattii* confers resistance to 5FC [103]. It has been reported that DNA mismatch repair confers an elevated mutation rate also enables rapid resistance to 5FC in *C. gattii* [104]. Through analysis of whole genomic sequence from 16 independent isolates, mutations associated with 5FC resistance were identified. These mutations were found in the known resistance genes, such as *FUR1* and *FCY2*, and *UXS1*, gene associated with alterations in capsule production and nucleotide metabolism [104]. Furthermore, multiple 5FC-resistant strains lacked mutations in any genes already known to cause 5FC resistance, indicating unknown mechanisms are responsible for resistance to 5FC in *C. gattii*.

## Conclusions

Initially considered as a variety of *C. neoformans*, *C. gattii* has since been elevated to a separate species, and has been grouped into several major molecular types based on genetic diversity. Given the close relationship between *C. gattii* and *C. neoformans*, the two species exhibit differences in patient predisposition, preferred body sites of infection, and environmental niche. *C. gattii* is an emerging fungal pathogen that causes lung and CNS infection in individuals both with and without apparently immune defects worldwide. It is valuable to understand the epidemiologic and pathogenic features of *C. gattii*. In this context, I have compiled a summary of the reported virulence factors in *C. gattii*. While several virulence factors, including capsule, melanin, and biofilm formation, have been widely studied in *C. neoformans*, with their regulatory mechanisms well-established to enhance fungal virulence, the precise contribution of these features to *C. gattii* infections remains enigmatic. Further investigation into how these pathogenic traits interplay in *C. gattii* infections is crucial for advancing our understanding of this emerging pathogen.

The clinical outcome of cryptococcosis caused by *C. gattii* is associated with early and accuracy of diagnosis, the host’s immune response, and the efficacy of the adopted treatment strategies. However, the prolonged latent period and the absence of specific symptoms associated with *C. gattii* infection pose challenges to achieving early and accurate diagnoses. Notably, *C. gattii* has the capability to infect even healthy individuals, suggesting a distinct host immune response pattern compared to *C. neoformans* infections. One of the host-dependent risk factors implicated in *C. gattii* infection is the GM-CSF neutralizing antibodies. This underscores the importance of investigating the immunomodulatory properties of *C. gattii* infections. To gain insights into these aspects, understanding patient status and intensifying research efforts are needed to elucidate the immunological interplay between *C. gattii* and its hosts. Such endeavors will be instrumental in refining diagnostic approaches, optimizing treatment strategies, and ultimately improving patient outcomes.

The development of antifungal drug treatment trials specific to *C. gattii* infections are important for the clinical management of this pathogen. Given the emergence of *C. gattii* as a public threat, it is imperative to establish large-scale, international collaborative networks that integrate expertise across various disciplines, including ecology, epidemiology, molecular biology, and clinical practices. These collaborative efforts will facilitate the understanding of biology, transmission dynamics, and host-*C. gattii* interactions, thereby enabling the design of more precise and effective therapeutic interventions.

**Key learning points**

**Epidemiology of *C. gattii***

The majority of *C. gattii*-infected patients are immunocompetent and apparently healthy individuals. GM-CSF autoantibodies in “apparently immunocompetent” patients significantly contribute to *C. gattii* infection.

**Fungal pathogenicity**

*Cryptococcus gattii* shares conserved virulence determinants, including capsule, melanin, and thermotolerance, biofilm production, titan cell formation, and CO_2_ tolerance, with *C. neoformans*. However, there are some distinct divergences between the two species. The conserved signaling pathways, such as the cyclic AMP/protein kinase A (cAMP/PKA), high osmolarity glycerol response (HOG), calcineurin, and cell wall integrity (CWI) pathways.

**Immunomodulatory strategies of *C. gattii***

*C. gattii* exhibits distinct immunomodulatory strategies compared to *C. neoformans*. *C. gattii* preferentially induces pro-inflammatory cytokine responses (IL-1β, TNF-α, IL-6) and Th17/IL-22 polarization while impairing neutrophil recruitment. Additionally, the prevalence of GM-CSF-neutralizing autoantibodies in immunocompetent hosts constitutes a critical susceptibility factor for *C. gattii* infection.

**Antifungal resistance pattern in *C. gattii***

The most important treatment options for cryptococcosis are fluconazole, amphotericin B, and flucytosine. In general, *C. gattii* shows less susceptible to azole drugs, particularly to fluconazole and voriconazole, compared to *C. neoformans*. Erg11 and ABC transporter contribute to the azole drug resistance observed in *C. gattii*.

**Five key papers in the field**

1. Stott KE, Loyse A, Jarvis JN, Alufandika M, Harrison TS, Mwandumba HC, et al. Cryptococcal meningoencephalitis: time for action. Lancet Infect Dis. 2021;21(9):e259–e71. Epub 2021/04/20. https://doi.org/10.1016/S1473-3099(20)30771-4. PubMed PMID: 33872594.
2. Galanis E, MacDougall L, Rose C, Chen SC, Oltean HN, Cieslak PR, et al. Predictors of *Cryptococcus gattii* clinical presentation and outcome: An international study. Clin Infect Dis. 2025. Epub 2025/01/08. https://doi.org/10.1093/cid/ciae640. PubMed PMID: 39774783.
3. Huang Y, Zang X, Yang C, Deng H, Ma X, Xie M, et al. Gene, virulence and related regulatory mechanisms in *Cryptococcus gattii*. Acta Biochim Biophys Sin (Shanghai). 2022;54(5):593–603. Epub 2022/05/21. https://doi.org/10.3724/abbs.2022029. PubMed PMID: 35593469; PubMed Central PMCID: PMCPMC9828318.
4. Saidykhan L, Onyishi CU, May RC. The *Cryptococcus gattii* species complex: Unique pathogenic yeasts with understudied virulence mechanisms. PLoS Negl Trop Dis. 2022;16(12):e0010916. Epub 2022/12/16. https://doi.org/10.1371/journal.pntd.0010916. PubMed PMID: 36520688; PubMed Central PMCID: PMCPMC9754292.
5. Chen SC, Meyer W, Sorrell TC. *Cryptococcus gattii* infections. Clin Microbiol Rev. 2014;27(4):980–1024. Epub 2014/10/04. https://doi.org/10.1128/CMR.00126-13. PubMed PMID: 25278580; PubMed Central PMCID: PMCPMC4187630.

## Abbreviations

APCs: antigen-presenting cells
cAMP: cyclic AMP
CGSC: *C. gattii* species complex
DCs: dendritic cells
GM-CSF: granulocyte-macrophage colony-stimulating factor
HOG: high osmolarity glycerol response
MAPK: mitogen-activated protein kinase
PBMCs: peripheral blood mononuclear cells
PKA: protein kinase A

## References

[1] Denning DW. Global incidence and mortality of severe fungal disease. Lancet Infect Dis. 2024;24(7):e428–38. doi: 10.1016/S1473-3099(23)00692-8 . 38224705

[2] Stott KE, Loyse A, Jarvis JN, Alufandika M, Harrison TS, Mwandumba HC, et al. Cryptococcal meningoencephalitis: time for action. Lancet Infect Dis. 2021;21(9):e259–71. doi: 10.1016/S1473-3099(20)30771-4 . 33872594

[3] Galanis E, MacDougall L, Rose C, Chen SCA, Oltean HN, Cieslak PR, et al. Predictors of *Cryptococcus gattii* clinical presentation and outcome: an international study. Clin Infect Dis. 2025;80(5):1088–94. doi: 10.1093/cid/ciae640 . 39774783 PMC12135907

[4] MacDougall L, Kidd SE, Galanis E, Mak S, Leslie MJ, Cieslak PR, et al. Spread of *Cryptococcus gattii* in British Columbia, Canada, and detection in the Pacific Northwest, USA. Emerg Infect Dis. 2007;13(1):42–50. doi: 10.3201/eid1301.060827 . 17370514 PMC2725832

[5] Rajasingham R, Govender NP, Jordan A, Loyse A, Shroufi A, Denning DW, et al. The global burden of HIV-associated cryptococcal infection in adults in 2020: a modelling analysis. Lancet Infect Dis. 2022;22(12):1748–55. doi: 10.1016/S1473-3099(22)00499-6 . 36049486 PMC9701154

[6] Montoya MC, Magwene PM, Perfect JR. Associations between *Cryptococcus* genotypes, phenotypes, and clinical parameters of human disease: a review. J Fungi (Basel). 2021;7(4):260. doi: 10.3390/jof7040260 . 33808500 PMC8067209

[7] Zang X, Ke W, Huang Y, Yang C, Song J, Deng H, et al. Virulence profiling of *Cryptococcus gattii* isolates in China: insights from a multi-center study. Microbiol Spectr. 2023;11(6):e0244323. doi: 10.1128/spectrum.02443-23 . 37905820 PMC10714995

[8] Zang X, Ke W, Wang L, Wu H, Huang Y, Deng H, et al. Molecular epidemiology and microbiological characteristics of *Cryptococcus gattii* VGII isolates from China. PLoS Negl Trop Dis. 2022;16(2):e0010078. doi: 10.1371/journal.pntd.0010078 . 35196319 PMC8901052

[9] Diaz JH. The disease ecology, epidemiology, clinical manifestations, and management of emerging *Cryptococcus gattii* complex infections. Wilderness Environ Med. 2020;31(1):101–9. doi: 10.1016/j.wem.2019.10.004 . 31813737

[10] Bielska E, May RC. What makes *Cryptococcus gattii* a pathogen? FEMS Yeast Res. 2016;16(1):fov106. doi: 10.1093/femsyr/fov106 . 26614308

[11] Kwon-Chung KJ, Saijo T. Is *Cryptococcus gattii* a primary pathogen? J Fungi (Basel). 2015;1(2):154–67. doi: 10.3390/jof1020154 . 27795955 PMC5084617

[12] Huang Y, Zang X, Yang C, Deng H, Ma X, Xie M, et al. Gene, virulence and related regulatory mechanisms in *Cryptococcus gattii*. Acta Biochim Biophys Sin (Shanghai). 2022;54(5):593–603. doi: 10.3724/abbs.2022029 . 35593469 PMC9828318

[13] Farrer RA, Desjardins CA, Sakthikumar S, Gujja S, Saif S, Zeng Q, et al. Genome evolution and innovation across the four major lineages of *Cryptococcus gattii*. mBio. 2015;6(5):e00868-15. doi: 10.1128/mBio.00868-15 . 26330512 PMC4556806

[14] Firacative C, Duan S, Meyer W. Galleria mellonella model identifies highly virulent strains among all major molecular types of *Cryptococcus gattii*. PLoS One. 2014;9(8):e105076. doi: 10.1371/journal.pone.0105076 . 25133687 PMC4136835

[15] Thompson GR 3rd, Albert N, Hodge G, Wilson MD, Sykes JE, Bays DJ, et al. Phenotypic differences of *Cryptococcus* molecular types and their implications for virulence in a *Drosophila* model of infection. Infect Immun. 2014;82(7):3058–65. doi: 10.1128/IAI.01805-14 . 24799631 PMC4097627

[16] Fraser JA, Giles SS, Wenink EC, Geunes-Boyer SG, Wright JR, Diezmann S, et al. Same-sex mating and the origin of the Vancouver Island *Cryptococcus gattii* outbreak. Nature. 2005;437(7063):1360–4. doi: 10.1038/nature04220 . 16222245

[17] Sellers B, Hall P, Cine-Gowdie S, Hays AL, Patel K, Lockhart SR, et al. *Cryptococcus gattii*: an emerging fungal pathogen in the Southeastern United States. Am J Med Sci. 2012;343(6):510–1. doi: 10.1097/MAJ.0b013e3182464bc7 . 22314106 PMC11904609

[18] Casadevall A, Coelho C, Cordero RJB, Dragotakes Q, Jung E, Vij R, et al. The capsule of *Cryptococcus neoformans*. Virulence. 2019;10(1):822–31. doi: 10.1080/21505594.2018.1431087 . 29436899 PMC6779390

[19] Saidykhan L, Onyishi CU, May RC. The *Cryptococcus gattii* species complex: Unique pathogenic yeasts with understudied virulence mechanisms. PLoS Negl Trop Dis. 2022;16(12):e0010916. doi: 10.1371/journal.pntd.0010916 . 36520688 PMC9754292

[20] Urai M, Kaneko Y, Ueno K, Okubo Y, Aizawa T, Fukazawa H, et al. Evasion of innate immune responses by the highly virulent *Cryptococcus gattii* by altering capsule glucuronoxylomannan structure. Front Cell Infect Microbiol. 2016;5:101. doi: 10.3389/fcimb.2015.00101 . 26779451 PMC4701946

[21] Ueno K, Otani Y, Yanagihara N, Urai M, Nagamori A, Sato-Fukushima M, et al. *Cryptococcus gattii* evades CD11b-mediated fungal recognition by coating itself with capsular polysaccharides. Eur J Immunol. 2021;51(9):2281–95. doi: 10.1002/eji.202049042 . 33728652

[22] Carneiro HCS, Bastos RW, Ribeiro NQ, Gouveia-Eufrasio L, Costa MC, Magalhães TFF, et al. Hypervirulence and cross-resistance to a clinical antifungal are induced by an environmental fungicide in *Cryptococcus gattii*. Sci Total Environ. 2020;740:140135. doi: 10.1016/j.scitotenv.2020.140135 . 32927573

[23] de Sousa HR, de Oliveira GP Jr, Frazão S de O, Gorgonha KC de M, Rosa CP, Garcez EM, et al. Faster *Cryptococcus* melanization increases virulence in experimental and human cryptococcosis. J Fungi (Basel). 2022;8(4):393. doi: 10.3390/jof8040393 . 35448624 PMC9029458

[24] Brilhante RSN, Rocha MG da, Oliveira JS de, Pereira-Neto WA, Guedes GM de M, Cordeiro R de A, et al. *Cryptococcus neoformans*/*Cryptococcus gattii* species complex melanized by epinephrine: Increased yeast survival after amphotericin B exposure. Microb Pathog. 2020;143:104123. doi: 10.1016/j.micpath.2020.104123 . 32169493

[25] Chen SC-A, Meyer W, Sorrell TC. *Cryptococcus gattii* infections. Clin Microbiol Rev. 2014;27(4):980–1024. doi: 10.1128/CMR.00126-13 . 25278580 PMC4187630

[26] Upadhya R, Lam WC, Maybruck B, Specht CA, Levitz SM, Lodge JK. Induction of protective immunity to cryptococcal infection in mice by a heat-killed, chitosan-deficient strain of *Cryptococcus neoformans*. mBio. 2016;7(3):e00547-16. doi: 10.1128/mBio.00547-16 . 27165801 PMC4959652

[27] Farrer RA, Ford CB, Rhodes J, Delorey T, May RC, Fisher MC, et al. Transcriptional heterogeneity of *Cryptococcus gattii* VGII compared with non-VGII lineages underpins key pathogenicity pathways. mSphere. 2018;3(5):e00445-18. doi: 10.1128/mSphere.00445-18 . 30355668 PMC6200987

[28] Lam WC, Upadhya R, Specht CA, Ragsdale AE, Hole CR, Levitz SM, et al. Chitosan biosynthesis and virulence in the human fungal pathogen *Cryptococcus gattii*. mSphere. 2019;4(5):e00644-19. doi: 10.1128/mSphere.00644-19 . 31597720 PMC6796976

[29] Tavares ER, Gionco B, Morguette AEB, Andriani GM, Morey AT, do Carmo AO, et al. Phenotypic characteristics and transcriptome profile of *Cryptococcus gattii* biofilm. Sci Rep. 2019;9(1):6438. doi: 10.1038/s41598-019-42896-2 . 31015652 PMC6478838

[30] Santi L, Berger M, Guimarães JA, Calegari-Alves YP, Vainstein MH, Yates JR 3rd, et al. Proteomic profile of *Cryptococcus gattii* biofilm: metabolic shift and the potential activation of electron chain transport. J Proteomics. 2024;290:105022. doi: 10.1016/j.jprot.2023.105022 . 37838096

[31] Ferrareze PAG, Streit RSA, Santos PRD, Santos FMD, Almeida RMC de, Schrank A, et al. Transcriptional analysis allows genome reannotation and reveals that *Cryptococcus gattii* VGII undergoes nutrient restriction during infection. Microorganisms. 2017;5(3):49. doi: 10.3390/microorganisms5030049 . 28832534 PMC5620640

[32] Benaducci T, Sardi J de CO, Lourencetti NMS, Scorzoni L, Gullo FP, Rossi SA, et al. Virulence of *Cryptococcus* sp. biofilms in vitro and in vivo using *Galleria mellonella* as an alternative model. Front Microbiol. 2016;7:290. doi: 10.3389/fmicb.2016.00290 . 27014214 PMC4783715

[33] Beardsley J, Dao A, Keighley C, Garnham K, Halliday C, Chen SC-A, et al. What’s new in *Cryptococcus gattii*: from bench to bedside and beyond. J Fungi (Basel). 2022;9(1):41. doi: 10.3390/jof9010041 . 36675862 PMC9865494

[34] Okagaki LH, Strain AK, Nielsen JN, Charlier C, Baltes NJ, Chrétien F, et al. Cryptococcal cell morphology affects host cell interactions and pathogenicity. PLoS Pathog. 2010;6(6):e1000953. doi: 10.1371/journal.ppat.1000953 . 20585559 PMC2887476

[35] Zaragoza O, García-Rodas R, Nosanchuk JD, Cuenca-Estrella M, Rodríguez-Tudela JL, Casadevall A. Fungal cell gigantism during mammalian infection. PLoS Pathog. 2010;6(6):e1000945. doi: 10.1371/journal.ppat.1000945 . 20585557 PMC2887474

[36] Fernandes KE, Fraser JA, Carter DA. Lineages derived from *Cryptococcus neoformans* type strain H99 support a link between the capacity to be pleomorphic and virulence. mBio. 2022;13(2):e0028322. doi: 10.1128/mbio.00283-22 . 35258331 PMC9040854

[37] García-Rodas R, de Oliveira HC, Trevijano-Contador N, Zaragoza O. Cryptococcal titan cells: when yeast cells are all grown up. Curr Top Microbiol Immunol. 2019;422:101–20. doi: 10.1007/82_2018_145 . 30406867

[38] Fernandes KE, Brockway A, Haverkamp M, Cuomo CA, van Ogtrop F, Perfect JR, et al. Phenotypic variability correlates with clinical outcome in *Cryptococcus* isolates obtained from Botswanan HIV/AIDS patients. mBio. 2018;9(5):e02016-18. doi: 10.1128/mBio.02016-18 . 30352938 PMC6199498

[39] Dyląg M, Colón-Reyes RJ, Loperena-Álvarez Y, Kozubowski L. Establishing minimal conditions sufficient for the development of Titan-like cells in *Cryptococcus neoformans*/*gattii* species complex. Pathogens. 2022;11(7):768. doi: 10.3390/pathogens11070768 . 35890013 PMC9322185

[40] Saidykhan L, Correia J, Romanyuk A, Peacock AFA, Desanti GE, Taylor-Smith L, et al. An in vitro method for inducing titan cells reveals novel features of yeast-to-titan switching in the human fungal pathogen *Cryptococcus gattii*. PLoS Pathog. 2022;18(8):e1010321. doi: 10.1371/journal.ppat.1010321 . 35969643 PMC9426920

[41] Kwon-Chung KJ, Fraser JA, Doering TL, Wang Z, Janbon G, Idnurm A, et al. *Cryptococcus neoformans* and *Cryptococcus gattii*, the etiologic agents of cryptococcosis. Cold Spring Harb Perspect Med. 2014;4(7):a019760. doi: 10.1101/cshperspect.a019760 . 24985132 PMC4066639

[42] Meng Y, Zhang C, Yi J, Zhou Z, Fa Z, Zhao J, et al. Deubiquitinase Ubp5 is required for the growth and pathogenicity of *Cryptococcus gattii*. PLoS One. 2016;11(4):e0153219. doi: 10.1371/journal.pone.0153219 . 27049762 PMC4822882

[43] Odom A, Muir S, Lim E, Toffaletti DL, Perfect J, Heitman J. Calcineurin is required for virulence of *Cryptococcus neoformans*. EMBO J. 1997;16(10):2576–89. doi: 10.1093/emboj/16.10.2576 . 9184205 PMC1169869

[44] Chen Y-L, Lehman VN, Lewit Y, Averette AF, Heitman J. Calcineurin governs thermotolerance and virulence of *Cryptococcus gattii*. G3 (Bethesda). 2013;3(3):527–39. doi: 10.1534/g3.112.004242 . 23450261 PMC3583459

[45] Chadwick BJ, Ristow LC, Xie X, Krysan DJ, Lin X. Discovery of CO_2_ tolerance genes associated with virulence in the fungal pathogen *Cryptococcus neoformans*. Nat Microbiol. 2024;9(10):2684–95. doi: 10.1038/s41564-024-01792-w . 39232204 PMC12883045

[46] Krysan DJ, Zhai B, Beattie SR, Misel KM, Wellington M, Lin X. Host carbon dioxide concentration is an independent stress for *Cryptococcus neoformans* that affects virulence and antifungal susceptibility. mBio. 2019;10(4):e01410-19. doi: 10.1128/mBio.01410-19 . 31266878 PMC6606813

[47] Jezewski AJ, Ristow LC, Krysan DJ. Carbon dioxide potentiates flucytosine susceptibility in *Cryptococcus neoformans*. Microbiol Spectr. 2023;11(2):e0478322. doi: 10.1128/spectrum.04783-22 . 36719209 PMC10101005

[48] Woith E, Fuhrmann G, Melzig MF. Extracellular vesicles-connecting kingdoms. Int J Mol Sci. 2019;20(22):5695. doi: 10.3390/ijms20225695 . 31739393 PMC6888613

[49] de Oliveira HC, Castelli RF, Reis FCG, Rizzo J, Rodrigues ML. Pathogenic delivery: the biological roles of cryptococcal extracellular vesicles. Pathogens. 2020;9(9):754. doi: 10.3390/pathogens9090754 . 32948010 PMC7557404

[50] Reis FCG, Borges BS, Jozefowicz LJ, Sena BAG, Garcia AWA, Medeiros LC, et al. A novel protocol for the isolation of fungal extracellular vesicles reveals the participation of a putative scramblase in polysaccharide export and capsule construction in *Cryptococcus gattii*. mSphere. 2019;4(2):e00080-19. doi: 10.1128/mSphere.00080-19 . 30894430 PMC6429041

[51] Reis FCG, Costa JH, Honorato L, Nimrichter L, Fill TP, Rodrigues ML. Small molecule analysis of extracellular vesicles produced by *Cryptococcus gattii*: identification of a tripeptide controlling cryptococcal infection in an invertebrate host model. Front Immunol. 2021;12:654574. doi: 10.3389/fimmu.2021.654574 . 33796117 PMC8008140

[52] Bielska E, Sisquella MA, Aldeieg M, Birch C, O’Donoghue EJ, May RC. Pathogen-derived extracellular vesicles mediate virulence in the fatal human pathogen *Cryptococcus gattii*. Nat Commun. 2018;9(1):1556. doi: 10.1038/s41467-018-03991-6 . 29674675 PMC5908794

[53] Maeng S, Ko Y-J, Kim G-B, Jung K-W, Floyd A, Heitman J, et al. Comparative transcriptome analysis reveals novel roles of the Ras and cyclic AMP signaling pathways in environmental stress response and antifungal drug sensitivity in *Cryptococcus neoformans*. Eukaryot Cell. 2010;9(3):360–78. doi: 10.1128/EC.00309-09 . 20097740 PMC2837985

[54] Caza M, Kronstad JW. The cAMP/protein kinase a pathway regulates virulence and adaptation to host conditions in *Cryptococcus neoformans*. Front Cell Infect Microbiol. 2019;9:212. doi: 10.3389/fcimb.2019.00212 . 31275865 PMC6592070

[55] Hicks JK, Heitman J. Divergence of protein kinase A catalytic subunits in *Cryptococcus neoformans* and *Cryptococcus gattii* illustrates evolutionary reconfiguration of a signaling cascade. Eukaryot Cell. 2007;6(3):413–20. doi: 10.1128/EC.00213-06 . 17189488 PMC1828938

[56] Bahn Y-S, Kojima K, Cox GM, Heitman J. Specialization of the HOG pathway and its impact on differentiation and virulence of *Cryptococcus neoformans*. Mol Biol Cell. 2005;16(5):2285–300. doi: 10.1091/mbc.e04-11-0987 . 15728721 PMC1087235

[57] Jung K-W, Strain AK, Nielsen K, Jung K-H, Bahn Y-S. Two cation transporters Ena1 and Nha1 cooperatively modulate ion homeostasis, antifungal drug resistance, and virulence of *Cryptococcus neoformans* via the HOG pathway. Fungal Genet Biol. 2012;49(4):332–45. doi: 10.1016/j.fgb.2012.02.001 . 22343280 PMC3319253

[58] So Y-S, Jang J, Park G, Xu J, Olszewski MA, Bahn Y-S. Sho1 and Msb2 play complementary but distinct roles in stress responses, sexual differentiation, and pathogenicity of *Cryptococcus neoformans*. Front Microbiol. 2018;9:2958. doi: 10.3389/fmicb.2018.02958 . 30564211 PMC6288190

[59] Huang Y-M, Tao X-H, Xu D-F, Yu Y, Teng Y, Xie W-Q, et al. HOG1 has an essential role in the stress response, virulence and pathogenicity of *Cryptococcus gattii*. Exp Ther Med. 2021;21(5):476. doi: 10.3892/etm.2021.9907 . 33767771 PMC7976431

[60] Bahn Y-S, Jung K-W. Stress signaling pathways for the pathogenicity of *Cryptococcus*. Eukaryot Cell. 2013;12(12):1564–77. doi: 10.1128/EC.00218-13 . 24078305 PMC3889573

[61] Lee D, Jang E-H, Lee M, Kim S-W, Lee Y, Lee K-T, et al. Unraveling melanin biosynthesis and signaling networks in *Cryptococcus neoformans*. mBio. 2019;10(5):e02267-19. doi: 10.1128/mBio.02267-19 . 31575776 PMC6775464

[62] Kozubowski L, Lee SC, Heitman J. Signalling pathways in the pathogenesis of *Cryptococcus*. Cell Microbiol. 2009;11(3):370–80. doi: 10.1111/j.1462-5822.2008.01273.x . 19170685 PMC3310389

[63] Motta H, Catarina Vieira Reuwsaat J, Daidrê Squizani E, da Silva Camargo M, Wichine Acosta Garcia A, Schrank A, et al. The small heat shock protein Hsp12.1 has a major role in the stress response and virulence of *Cryptococcus gattii*. Fungal Genet Biol. 2023;165:103780. doi: 10.1016/j.fgb.2023.103780 . 36780981

[64] Chang YC, Khanal Lamichhane A, Bradley J, Rodgers L, Ngamskulrungroj P, Kwon-Chung KJ. Differences between *Cryptococcus neoformans* and *Cryptococcus gattii* in the molecular mechanisms governing utilization of D-amino acids as the sole nitrogen source. PLoS One. 2015;10(7):e0131865. doi: 10.1371/journal.pone.0131865 . 26132227 PMC4489021

[65] Ren P, Chaturvedi V, Chaturvedi S. Carbon dioxide is a powerful inducer of monokaryotic hyphae and spore development in *Cryptococcus gattii* and carbonic anhydrase activity is dispensable in this dimorphic transition. PLoS One. 2014;9(12):e113147. doi: 10.1371/journal.pone.0113147 . 25478697 PMC4257545

[66] Rodrigues J, Fonseca FL, Schneider RO, Godinho RM da C, Firacative C, Maszewska K, et al. Pathogenic diversity amongst serotype C VGIII and VGIV *Cryptococcus gattii* isolates. Sci Rep. 2015;5:11717. doi: 10.1038/srep11717 . 26153364 PMC4495446

[67] Schneider R de O, Fogaça N de SS, Kmetzsch L, Schrank A, Vainstein MH, Staats CC. Zap1 regulates zinc homeostasis and modulates virulence in *Cryptococcus gattii*. PLoS One. 2012;7(8):e43773. doi: 10.1371/journal.pone.0043773 . 22916306 PMC3423376

[68] Schneider R de O, Diehl C, Dos Santos FM, Piffer AC, Garcia AWA, Kulmann MIR, et al. Effects of zinc transporters on *Cryptococcus gattii* virulence. Sci Rep. 2015;5:10104. doi: 10.1038/srep10104 . 25951314 PMC4423424

[69] Garcia AWA, Kinskovski UP, Diehl C, Reuwsaat JCV, Motta de Souza H, Pinto HB, et al. Participation of Zip3, a ZIP domain-containing protein, in stress response and virulence in *Cryptococcus gattii*. Fungal Genet Biol. 2020;144:103438. doi: 10.1016/j.fgb.2020.103438 . 32738289

[70] Ngamskulrungroj P, Price J, Sorrell T, Perfect JR, Meyer W. *Cryptococcus gattii* virulence composite: candidate genes revealed by microarray analysis of high and less virulent Vancouver island outbreak strains. PLoS One. 2011;6(1):e16076. doi: 10.1371/journal.pone.0016076 . 21249145 PMC3020960

[71] Yang C, Shen W, Wang L, Zang X, Huang Y, Deng H, et al. *Cryptococcus gattii* strains with a high phagocytosis phenotype by macrophages display high pathogenicity at the early stage of infection in vivo. Acta Biochim Biophys Sin (Shanghai). 2024;56(2):291–303. doi: 10.3724/abbs.2023250 . 37885429 PMC10984874

[72] Shoham S, Huang C, Chen JM, Golenbock DT, Levitz SM. Toll-like receptor 4 mediates intracellular signaling without TNF-alpha release in response to *Cryptococcus neoformans* polysaccharide capsule. J Immunol. 2001;166(7):4620–6. doi: 10.4049/jimmunol.166.7.4620 . 11254720

[73] Fonseca FL, Nohara LL, Cordero RJB, Frases S, Casadevall A, Almeida IC, et al. Immunomodulatory effects of serotype B glucuronoxylomannan from *Cryptococcus gattii* correlate with polysaccharide diameter. Infect Immun. 2010;78(9):3861–70. doi: 10.1128/IAI.00111-10 . 20547742 PMC2937472

[74] Huang H-R, Li F, Han H, Xu X, Li N, Wang S, et al. Dectin-3 recognizes glucuronoxylomannan of *Cryptococcus neoformans* serotype AD and *Cryptococcus gattii* serotype B to initiate host defense against cryptococcosis. Front Immunol. 2018;9:1781. doi: 10.3389/fimmu.2018.01781 . 30131805 PMC6090260

[75] Jia X-M, Tang B, Zhu L-L, Liu Y-H, Zhao X-Q, Gorjestani S, et al. CARD9 mediates Dectin-1-induced ERK activation by linking Ras-GRF1 to H-Ras for antifungal immunity. J Exp Med. 2014;211(11):2307–21. doi: 10.1084/jem.20132349 . 25267792 PMC4203953

[76] Huston SM, Li SS, Stack D, Timm-McCann M, Jones GJ, Islam A, et al. *Cryptococcus gattii* is killed by dendritic cells, but evades adaptive immunity by failing to induce dendritic cell maturation. J Immunol. 2013;191(1):249–61. doi: 10.4049/jimmunol.1202707 . 23740956

[77] Huston SM, Ngamskulrungroj P, Xiang RF, Ogbomo H, Stack D, Li SS, et al. *Cryptococcus gattii* capsule blocks surface recognition required for dendritic cell maturation independent of internalization and antigen processing. J Immunol. 2016;196(3):1259–71. doi: 10.4049/jimmunol.1501089 . 26740109

[78] Jamil K, Polyak MJ, Feehan DD, Surmanowicz P, Stack D, Li SS, et al. Phagosomal F-actin retention by *Cryptococcus gattii* induces dendritic cell immunoparalysis. mBio. 2020;11(6):e01821-20. doi: 10.1128/mBio.01821-20 . 33234684 PMC7701985

[79] Schoffelen T, Illnait-Zaragozi M-T, Joosten LAB, Netea MG, Boekhout T, Meis JF, et al. *Cryptococcus gattii* induces a cytokine pattern that is distinct from other cryptococcal species. PLoS One. 2013;8(1):e55579. doi: 10.1371/journal.pone.0055579 . 23383232 PMC3561320

[80] Piffer AC, Santos FMD, Thomé MP, Diehl C, Garcia AWA, Kinskovski UP, et al. Transcriptomic analysis reveals that mTOR pathway can be modulated in macrophage cells by the presence of cryptococcal cells. Genet Mol Biol. 2021;44(3):e20200390. doi: 10.1590/1678-4685-GMB-2020-0390 . 34352067 PMC8341293

[81] Holcomb ZE, Steinbrink JM, Zaas AK, Betancourt M, Tenor JL, Toffaletti DL, et al. Transcriptional profiles elucidate differential host responses to infection with *Cryptococcus neoformans* and *Cryptococcus gattii*. J Fungi (Basel). 2022;8(5):430. doi: 10.3390/jof8050430 . 35628686 PMC9143552

[82] Da Silva CA, Hartl D, Liu W, Lee CG, Elias JA. TLR-2 and IL-17A in chitin-induced macrophage activation and acute inflammation. J Immunol. 2008;181(6):4279–86. doi: 10.4049/jimmunol.181.6.4279 . 18768886 PMC2577310

[83] da Silva-Junior EB, Firmino-Cruz L, Guimarães-de-Oliveira JC, De-Medeiros JVR, de Oliveira Nascimento D, Freire-de-Lima M, et al. The role of toll-like receptor 9 in a murine model of *Cryptococcus gattii* infection. Sci Rep. 2021;11(1):1407. doi: 10.1038/s41598-021-80959-5 . 33446850 PMC7809259

[84] Wright L, Bubb W, Davidson J, Santangelo R, Krockenberger M, Himmelreich U, et al. Metabolites released by *Cryptococcus neoformans* var. *neoformans* and var. *gattii* differentially affect human neutrophil function. Microbes Infect. 2002;4(14):1427–38. doi: 10.1016/s1286-4579(02)00024-2 . 12475633

[85] Cheng P-Y, Sham A, Kronstad JW. *Cryptococcus gattii* isolates from the British Columbia cryptococcosis outbreak induce less protective inflammation in a murine model of infection than *Cryptococcus neoformans*. Infect Immun. 2009;77(10):4284–94. doi: 10.1128/IAI.00628-09 . 19635827 PMC2747943

[86] Angkasekwinai P, Sringkarin N, Supasorn O, Fungkrajai M, Wang Y-H, Chayakulkeeree M, et al. *Cryptococcus gattii* infection dampens Th1 and Th17 responses by attenuating dendritic cell function and pulmonary chemokine expression in the immunocompetent hosts. Infect Immun. 2014;82(9):3880–90. doi: 10.1128/IAI.01773-14 . 24980974 PMC4187835

[87] Wang S-Y, Lo Y-F, Shih H-P, Ho M-W, Yeh C-F, Peng J-J, et al. *Cryptococcus gattii* infection as the major clinical manifestation in patients with autoantibodies against granulocyte-macrophage colony-stimulating factor. J Clin Immunol. 2022;42(8):1730–41. doi: 10.1007/s10875-022-01341-2 . 35947322

[88] Iyer KR, Revie NM, Fu C, Robbins N, Cowen LE. Treatment strategies for cryptococcal infection: challenges, advances and future outlook. Nat Rev Microbiol. 2021;19(7):454–66. doi: 10.1038/s41579-021-00511-0 . 33558691 PMC7868659

[89] Chen YC, Chang SC, Shih CC, Hung CC, Luhbd KT, Pan YS, et al. Clinical features and in vitro susceptibilities of two varieties of *Cryptococcus neoformans* in Taiwan. Diagn Microbiol Infect Dis. 2000;36(3):175–83. doi: 10.1016/s0732-8893(99)00137-6 . 10729660

[90] Trilles L, Meyer W, Wanke B, Guarro J, Lazéra M. Correlation of antifungal susceptibility and molecular type within the *Cryptococcus neoformans*/*C. gattii* species complex. Med Mycol. 2012;50(3):328–32. doi: 10.3109/13693786.2011.602126 . 21859388

[91] Al-Odaini N, Li X-Y, Li B-K, Chen X-C, Huang C-Y, Lv C-Y, et al. In vitro antifungal susceptibility profiles of *Cryptococcus neoformans* var. *grubii* and *Cryptococcus gattii* clinical isolates in Guangxi, Southern China. Front Microbiol. 2021;12:708280. doi: 10.3389/fmicb.2021.708280 . 34447360 PMC8383296

[92] Carvajal SK, Melendres J, Escandón P, Firacative C. Reduced susceptibility to azoles in *Cryptococcus gattii* correlates with the substitution R258L in a substrate recognition site of the lanosterol 14-α-demethylase. Microbiol Spectr. 2023;11(4):e0140323. doi: 10.1128/spectrum.01403-23 . 37341584 PMC10434158

[93] Chowdhary A, Randhawa HS, Sundar G, Kathuria S, Prakash A, Khan Z, et al. In vitro antifungal susceptibility profiles and genotypes of 308 clinical and environmental isolates of *Cryptococcus neoformans* var. *grubii* and *Cryptococcus gattii* serotype B from north-western India. J Med Microbiol. 2011;60(Pt 7):961–7. doi: 10.1099/jmm.0.029025-0 . 21393452

[94] Trilles L, Fernández-Torres B, Lazéra M dos S, Wanke B, Guarro J. In vitro antifungal susceptibility of *Cryptococcus gattii*. J Clin Microbiol. 2004;42(10):4815–7. doi: 10.1128/JCM.42.10.4815-4817.2004 . 15472349 PMC522305

[95] Lin K-H, Lai Y-C, Lin Y-P, Ho M-W, Chen Y-C, Chung W-H. Antifungal susceptibility of the clinical and environmental strains of *Cryptococcus gattii* sensu lato in Taiwan. Mycoses. 2023;66(1):13–24. doi: 10.1111/myc.13520 . 35986599

[96] Firacative C, Escandón P. Antifungal susceptibility of clinical *Cryptococcus gattii* isolates from Colombia varies among molecular types. Med Mycol. 2021;59(11):1122–5. doi: 10.1093/mmy/myab041 . 34264298 PMC8757315

[97] Herkert PF, Hagen F, Pinheiro RL, Muro MD, Meis JF, Queiroz-Telles F. Ecoepidemiology of *Cryptococcus gattii* in developing countries. J Fungi (Basel). 2017;3(4):62. doi: 10.3390/jof3040062 . 29371578 PMC5753164

[98] Taverna CG, Arias BA, Firacative C, Vivot ME, Szusz W, Vivot W, et al. Genotypic diversity and antifungal susceptibility of clinical isolates of *Cryptococcus Gattii* species complex from Argentina. Mycopathologia. 2023;188(1–2):51–61. doi: 10.1007/s11046-022-00705-x . 36609823

[99] Campos Péret VA, Reis RCFM, Braga SFP, Benedetti MD, Caldas IS, Carvalho DT, et al. New miconazole-based azoles derived from eugenol show activity against *Candida* spp. and *Cryptococcus gattii* by inhibiting the fungal ergosterol biosynthesis. Eur J Med Chem. 2023;256:115436. doi: 10.1016/j.ejmech.2023.115436 . 37146343

[100] Xue X, Zang X, Xiao M, Wang L, Wu H, Ma X, et al. Significance of differential expression profiles of ABC transporters in azole susceptibility between *Cryptococcus gattii* VGI and VGII strains. Med Mycol. 2022;60(7):myac035. doi: 10.1093/mmy/myac035 . 35641230

[101] Scorzoni L, de Paula E Silva ACA, Marcos CM, Assato PA, de Melo WCMA, de Oliveira HC, et al. Antifungal therapy: new advances in the understanding and treatment of mycosis. Front Microbiol. 2017;8:36. doi: 10.3389/fmicb.2017.00036 . 28167935 PMC5253656

[102] Gast CE, Basso LR Jr, Bruzual I, Wong B. Azole resistance in *Cryptococcus gattii* from the Pacific Northwest: investigation of the role of ERG11. Antimicrob Agents Chemother. 2013;57(11):5478–85. doi: 10.1128/AAC.02287-12 . 23979758 PMC3811322

[103] Khanal Lamichhane A, Garraffo HM, Cai H, Walter PJ, Kwon-Chung KJ, Chang YC. A novel role of fungal type I myosin in regulating membrane properties and its association with D-amino acid utilization in *Cryptococcus gattii*. mBio. 2019;10(4):e01867-19. doi: 10.1128/mBio.01867-19 . 31455652 PMC6712397

[104] Billmyre RB, Applen Clancey S, Li LX, Doering TL, Heitman J. 5-Fluorocytosine resistance is associated with hypermutation and alterations in capsule biosynthesis in *Cryptococcus*. Nat Commun. 2020;11(1):127. Epub 2020/01/09. doi: 10.1038/s41467-019-13890-z ; PMCID: PMC6949227. 31913284 PMC6949227

